# Quantifying movement of optical structures during radiotherapy treatment for tumors near the orbita

**DOI:** 10.1016/j.phro.2025.100830

**Published:** 2025-08-30

**Authors:** Femke Vaassen, David Hofstede, Nikolina Birimac, Inge Compter, Marlies Granzier, Wouter van Elmpt, Catharina M.L. Zegers, Daniëlle B.P. Eekers

**Affiliations:** Department of Radiation Oncology (Maastro), GROW Research Institute for Oncology and Reproduction, Maastricht University Medical Centre+, Maastricht, the Netherlands

**Keywords:** Radiation therapy, Optical movement, Organs-at-risk, PRV margins

## Abstract

**Background and purpose:**

Movement of optical structures during radiotherapy for tumors near the orbita might affect the amount of radiation given and consequently the risk for side effects. The aim of this study was therefore to quantify motion of optical structures during radiotherapy.

**Materials and methods:**

Twenty brain tumor patients were retrospectively included, with planning computed tomography (CT)-scan (pCT) and five repeat-CT-scans (reCTs) without gazing instructions. Six optical structures were delineated bilaterally: lens, cornea, retina, lacrimal glands, macula, and optic nerves (ON). The ON was split in three subregions. The dice similarity coefficient (DSC), absolute distance (AD), and difference in 3D midpoint (ΔMP) were calculated between pCT and reCT. Planning risk volume (PRV)-margins and isotropic expansions to cover 95 % volume for 90 % patients were calculated. A dose-volume proof-of-principle was performed for a neurological and nasopharyngeal tumor.

**Results:**

Highest median ΔMP was found for the cornea: 1.9 mm. For ON subregions, highest median and 95th-percentile ΔMP was found for proximal intra-orbital ON: 1.3 mm and 3.1 mm. ON showed highest median AD: 3.0 mm (negative Z-direction). Open eyelid status resulted in statistically significant lower DSC for ON, intra-cranial ON, and proximal intra-orbital ON, and higher ΔMP for proximal intra-orbital ON. 1–4 mm isotropic expansions were needed for separate structures, dependent on typical movement range. Higher dose-differences were found for the neurological than the nasopharyngeal plan.

**Conclusions:**

The observed movement of optical structures indicated that a PRV-margin should be considered in clinical practice. Asking the patient to close their eyes during the treatment could decrease the movement.

## Highlights

• Highest movement of the center-of-mass midpoint was seen for the cornea.
• Intra-orbital optic nerve subregion moved more than the intra-cranial subregion.
• Closed eyelid status proved to be beneficial in decreasing optical movement.
• An isotropic planning margin of 4 mm could be incorporated for some structures.

## Graphical abstract

**Figure:**
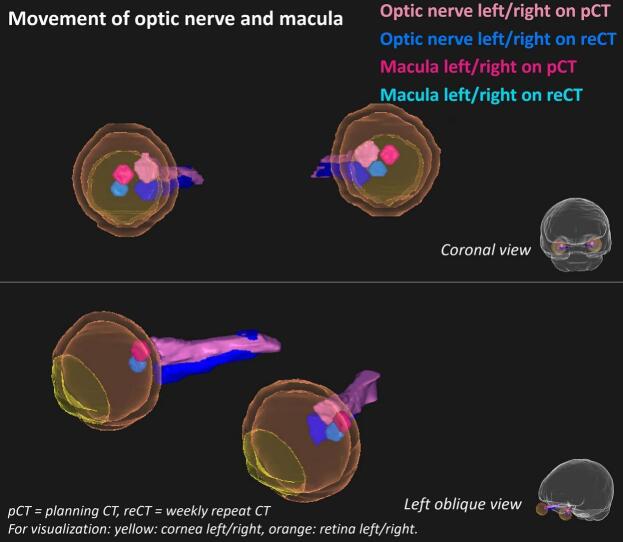

## 1 Introduction

Radiation treatment (RT) for tumors near the orbita, such as periorbital and nasopharynx sites, is challenging due to the complexity, sensitivity, and mobility of optical structures [1]. Radiation to the orbital region can result in side effects such as dry eyes, radiation retinopathy, vascular occlusion, or even permanent blindness [2,3]. Optical structures are delineated on the computed tomography (CT)-scan and during treatment planning the dose distribution is optimized to avoid the delivery of high doses to this region [4]. However, potential movement of these structures during treatment can lead to unintended radiation exposure.

Greater certainty in the position of optical structures during treatment fractions presents an opportunity for further treatment optimization. Studies suggest a need for planning risk volume (PRV)-margin expansion during treatment planning to account for optical movement over the course of treatment, but these focused on only one optical structure [[5], [6], [7]]. A technical feasibility study of one patient showed that a PRV-margin expansion for the lenses as well as for the optic nerves was necessary to encompass their various positions over the course of treatment [8]. These studies showed there is a need for margin expansion. However, also optical structures other than the optic nerves and lenses should be considered.

To determine if the movement and position of optical structures are significant for treatment planning and execution, it is important to understand the extent of the natural gaze extent and therefore movement of the optical structures that they display during treatment. This research aims to investigate the typical movement of several optical structures commonly deemed organs-at-risk (OARs): lens, cornea, retina, lacrimal glands, macula, and optic nerve (ON). This will be achieved by quantifying the movement of these structures over the course of RT, without using gazing instructions. The eyelid status − being open or closed − will be evaluated as an influential factor.

## 2 Materials and methods

### 2.1 Patient population and imaging

For this study, 20 brain tumor patients treated with intensity modulated proton therapy (IMPT; Mevion Hyperscan S250i) between November 2019 and November 2020 at Maastro, Maastricht, the Netherlands, were retrospectively analyzed. Patients’ characteristics were summarized in Supplementary Material Table S1. Patients received a pre-treatment (planning) CT-scan (pCT) and at least five weekly repeat CT-scans (reCTs), according to standard clinical practice at the time, with a slice thickness of 1 mm in supine position, without receiving gazing instructions (SOMATOM Drive or Confidence; Siemens, Erlangen). A total of 120 CT-scans were used for the analysis. Thermoplastic immobilization masks (Orfit, Wijnegem, Belgium) were used during CT-scanning and treatment. Patients had no underlying optic conditions such as a preorbital tumor or vision impairment. The Institutional Review Board approved this retrospective study with project number P0419.

### 2.2 Structure delineation

Six optical structures (lens, cornea, retina, lacrimal glands, macula, and optic nerves (ON)) were delineated bilaterally on each CT-scan in accordance with the EPTN contouring atlas guidelines in the Eclipse Treatment Planning System (Varian Medical Systems Inc, Palo Alto, California, USA) [9]. The ON was split into three subregions (intra-cranial, distal intra-orbital, and proximal intra-orbital). The distal intra-orbital and proximal intra-orbital subregions were also considered together (intra-orbital). Both oculi were created as the sum of the retina including their inner volume and the cornea, because displacement observed in the oculi could indicate inaccuracies in image registration, delineation, or set-up. All structures were contoured by consensus of two delineators to assure accuracy and reliability. The left and right version of a structure were analyzed together, resulting in 11 structures in the final analysis.

### 2.3 Geometrical analysis of movement

The contouring of the structures on all pCT and reCTs allowed for the comparison of the position on the reCTs to the original position on the pCT. The reCTs were rigidly registered to the pCT within Eclipse. MATLAB2024b (The MathWorks Inc, Natick, Massachusetts, USA) was used for further analysis.

**Fig. 1:**
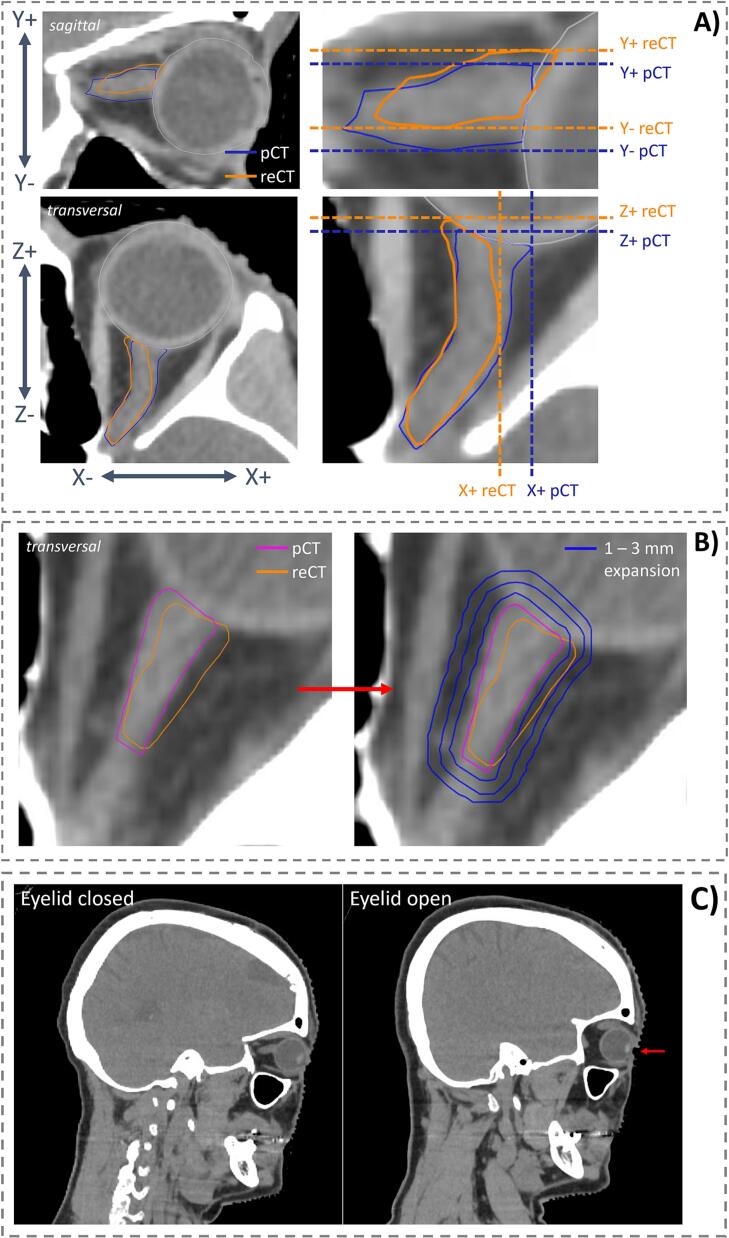
A) Visualization of the absolute distance (AD), shown here in the orbita for an optic nerve substructure. In both the positive and negative side of each direction (X, Y, and Z), the edges of the contours were determined, and the difference was calculated. A positive difference in either direction meant that the contour on the reCT was bigger than the contour on the pCT. B) Transversal visualization of 1, 2 and 3 mm isotropic expansion of a pCT structure (pink). The reCT structure (orange) falls within the 2 mm expanded pCT structure in this example. C) Comparison of eyelid status in two patients: The left image shows a patient with closed eyelids, while the right image depicts a patient with open eyelids. The red arrow indicates the eyelid opening, highlighting the anatomical difference that may influence optical structure movement during radiotherapy. Eyelid status was determined using a sagittal plane measurement tool with a predefined cutoff of 2 mm. (For interpretation of the references to colour in this figure legend, the reader is referred to the web version of this article.)

The dice similarity coefficient (DSC), the absolute distance (AD) between the two contours and the absolute difference in 3D midpoint position (ΔMP) were calculated between the pCT and each reCT contour. The AD was employed to quantify positional discrepancies by examining the opposite edge of the contours as depicted in Fig. 1A. In both the positive and negative side of each direction (X, Y, and Z), the difference between the edges of the contour was calculated for each direction and both sides. This resulted in six difference values per structure per reCT. ΔMP was also analyzed for the X, Y, and Z direction.

Additionally, each structure on the pCT was isotropically expanded with 1, 2, 3, 4 and 5 mm. The percentage of a structure on a reCT within the volume of the structure on the pCT, and within the isotropically expanded structure, was calculated (see Fig. 1B). The percentage of the volume being covered with the isotropic expansions for 90 % of patients was calculated thereafter.

The status of the eyelid per reCT, being open or closed, was included in the investigation as a confounding variable to see whether there was a relationship between this status and the extent of movement. The measurements were conducted in the sagittal plane and a cut-off value of 2 mm was used to decide if an eyelid was open or closed. An example of one patient with open and one with closed eyelids is shown in Fig. 1C.

### 2.4 Dose-volume analysis

To estimate the potential dose-volume influence of the movement of optical structures, a worst-case scenario was investigated for the patient with the largest optical movement. Two hypothetical tumor volumes were delineated on the pCT to simulate a neurological (33x1.8 Gy) and a nasopharyngeal (35x2Gy) tumor. Treatment planning was performed on the pCT according to clinical practice using Eclipse (Acuros 16.1.0, Varian Medical Systems). The registered optical structures from the reCTs were used to extract dose-volume histogram (DVH) parameters for each movement of the optical structures, and mean dose (D_mean_) and dose to 0.03 cm^3^ of the volume (D_0.03cm3_) were extracted.

### 2.5 Statistical analyses

The average DSC per patient was calculated by averaging the DSC per structure over the five reCTs per patient. To show the diversity in the patient group, the median (range) of these average DSC values was reported. For the distance-related metrics (AD and ΔMP), results were presented in two ways: 1) as the 95th-percentile over all reCTs, to show the maximum difference over all patients and all reCTs, excluding outliers, and 2) as the median (range) after averaging the five reCTs per patient, to show average difference in the patient group. To assess whether eyelid status had a significant impact on optical movement, a Mann-Whitney *U* test was used. A Bonferroni correction to account for multiple testing was applied. A p-value < 0.0045 (0.05/11) was considered statistically significant.

## 3 Results

### 3.1 Geometrical analysis

The oculus had a median DSC of 0.95 (0.91–0.97). The retina showed the next highest median DSC: 0.80 (0.70–0.90). Lowest median DSC was found for the macula: 0.46 (0.16–0.71). See Supplementary Material Table S2 for all DSC results.

Highest median ΔMP vector was found for the cornea (1.9 mm, range: 0.9–3.4 mm). Specifications per direction can be found in Table 1. Highest 95th-percentile ΔMP vector over all reCTs was found for the cornea (4.3 mm). Median and the 95th-percentile ΔMP vector of the oculus was 0.7 and 1.4 mm. For the ON subregions, highest median and 95th-percentile ΔMP vector was found for the proximal intra-orbital ON (1.3 mm (range: 0.7–2.7 mm), and 3.1 mm).

**Table 1:** Overview of absolute ΔMP results, presented in X, Y, and Z direction as well as the vector. Values are presented first as the 95th-percentile over all reCTs, to show the maximum difference in midpoint over all patients but excluding outliers. Secondly, results are presented as the median (range) after first averaging over the five reCTs per patient, to show average difference in midpoint in the patient group.

Structure	95th^-^percentile over all reCTs [mm]				Median (range) of average per patient [mm]			
	*X*	*Y*	*Z*	*Vector*	*X*	*Y*	*Z*	*Vector*
Cornea	2.5	3.8	1.5	4.3	0.0 (−2.9–1.8)	0.7 (−2.4–2.3)	0.1 (−0.9–1.2)	1.9 (0.9–3.4)
Lacrimal gland	1.1	2.5	1.4	2.8	0.0 (−2.1–1.1)	0.0 (−2.7–1.6)	0.1 (−1.4–1.4)	1.0 (0.4–3.1)
Lens	2.0	3.3	1.1	3.7	−0.2 (−2.1–2.1)	0.6 (−2.1–1.9)	0.2 (−0.7–1.0)	1.6 (0.8–3.3)
Macula	2.3	3.2	1.6	3.9	0.3 (−1.3–2.6)	−0.6 (−2.2–1.9)	0.0 (−1.2–1.5)	1.8 (0.8–3.3)
Oculus	0.9	1.1	0.8	1.4	0.0 (−0.5–1.1)	0.1 (−0.7–0.9)	0.1 (−0.8–0.9)	0.7 (0.2–1.4)
Optic nerve	1.2	1.3	1.8	2.2	0.1 (−1.1–1.2)	−0.2 (−1.0–0.9)	0.2 (−1.2–1.6)	1.1 (0.7–1.9)
Optic nerve intra-cranial	0.9	0.6	1.2	1.5	0.0 (−0.9–0.6)	−0.1 (−0.6–0.3)	−0.1 (−1.1–1.0)	0.6 (0.2–1.5)
Optic nerve intra-orbital	1.0	1.8	1.3	2.1	0.1 (−1.0–1.3)	−0.3 (−1.2–1.1)	0.1 (−1.2–1.0)	1.0 (0.4–1.8)
Optic nerve distal intra-orbital	0.9	0.8	1.5	1.9	−0.1 (−3.2–0.6)	−0.1 (−0.9–0.4)	0.0 (−3.9–0.8)	0.7 (0.3–5.0)
Optic nerve proximal intra-orbital	1.5	2.5	1.5	3.1	0.1 (−1.1–2.0)	−0.4 (−1.6–1.7)	0.0 (−1.0–1.3)	1.3 (0.7–2.7)
Retina	0.8	1.1	0.9	1.4	0.1 (−0.4–1.1)	0.0 (−0.8–0.9)	0.0 (−0.9–0.7)	0.7 (0.3–1.5)

ON presented with the highest median AD of 3.0 mm (range: 0.2–13.3 mm) in the negative Z-direction, see Table 2. Intra-orbital subregions of the ON showed larger AD compared with the intra-cranial subregion. E.g., for the negative Z-direction, median AD of the intra-orbital ON was 2.5 mm (range: 0.0–11.4 mm) compared to 0.7 mm (range: −0.5–2.9 mm) for the intra-cranial ON. AD greater than 1 mm was observed in 93 % of reCTs over all structures, and greater than 2 mm in 60 % of reCTs. Highest occurrence of greater than 2 mm was found for the lacrimal gland (84 %, see Supplementary Material Table S3).

**Table 2:** Overview of absolute difference (AD) results, presented in positive and negative X, Y, and Z direction. Values are presented first as the 95th-percentile over all reCTs, to show the maximum AD over all patients but excluding outliers. Secondly, results are presented as the median (range) after first averaging over the five reCTs per patient, to show average AD in the patient group.

Structure	95th^-^percentile over all reCTs [mm]						Median (range) of average per patient [mm]					
	*X-*	*X+*	*Y-*	*Y+*	*Z-*	*Z+*	*X-*	*X+*	*Y-*	*Y+*	*Z-*	*Z+*
Cornea	4.9	5.4	3.0	2.0	3.5	2.5	2.0 (−0.6–3.6)	1.9 (0.2–4.8)	0.3 (−3.0–1.4)	−1.2 (−4.6–2.6)	1.3 (0.3–3.0)	1.0 (0.3–2.8)
Lacrimal gland	2.7	3.2	2.0	2.0	5.5	4.8	1.2 (0.3–2.5)	1.2 (0.2–18.2)	0.0 (−5.2–1.8)	−0.2 (−1.6–2)	2.1 (0.4–6.6)	1.8 (0.2–5.6)
Lens	3.2	2.9	3.0	2.0	2.1	1.1	0.9 (−1.1–3.1)	1.2 (−1.0–3.5)	0.4 (−2.0–2.4)	−0.6 (−2.6–2.6)	0.8 (−0.5–2.3)	0.3 (−0.4–1.1)
Macula	2.9	2.3	1.0	2.0	1.1	1.5	0.9 (−1.0–3.2.0)	0.3 (−2.1–1.8)	−1.0 (−2.6–1.6)	0.7 (−2.4–1.8)	0.1 (−0.9–1.7)	0.4 (−1.4–1.9)
Oculus	3.2	3.6	2.0	1.0	3.8	3.7	1.6 (0.2–3.1)	1.4 (0.3–4.5)	0.2 (−1.0–1.6)	−0.7 (−1.0–1.2)	1.3 (0.4–3.9)	1.8 (0.6–3.7)
Optic nerve	6.2	9.1	1.0	2.0	11.9	20.7	1.4 (0.0–10.9)	1.4 (−1.1–14.2)	−0.3 (−1.8–1.4)	0.2 (−1.8–1.8)	3.0 (0.2–13.3)	1.6 (−0.7–22.8)
Optic nerve intra-cranial	1.8	2.4	1.0	1.0	2.9	2.2	0.4 (−0.5–2.1)	0.5 (−0.9–2.5)	0.0 (−1.0–1.0)	0.0 (−0.4–0.8)	0.7 (−0.5–2.9)	0.7 (−0.2–2.7)
Optic nerve intra-orbital	6.1	7.7	1.0	2.0	10.3	19.8	1.2 (0.0–6.4)	1.1 (−1.1–7.1)	−0.4 (−2.2–1.4)	0.2 (−2.2–2.2)	2.5 (0.0–11.4)	1.5 (−0.7–16.4)
Optic nerve distal intra-orbital	2.5	3.6	1.0	1.0	5.6	5.0	0.5 (−1.0–2.5)	0.5 (−0.7–3.4)	−0.3 (−2.0–0.6)	0.0 (−1.0–0.8)	0.9 (−0.5–5.9)	0.9 (−0.7–7.8)
Optic nerve proximal intra-orbital	3.4	2.6	1.0	2.0	8.2	7.4	1.0 (−0.2–4.2)	0.5 (−1.8–3.0)	−0.7 (−6.4–2.0)	0.4 (−2.4–2.2)	1.3 (−0.9–7.0)	1.0 (−0.7–7.8)
Retina	3.1	3.6	1.0	1.0	3.8	3.2	1.4 (0.2–3.1)	1.4 (0.2–4.5)	0.0 (−1.2–1.4)	−0.3 (−1.0–0.8)	1.2 (0.2–3.9)	1.5 (0.8–2.8)

After isotropic expansion on the pCT, the percentage of a reCT structure within both the original and expanded pCT volumes was calculated. The percentage of the volume being covered with the isotropic expansions for 90 % of patients and the specific expansion needed to cover 95 % volume was determined (see Table 3).

**Table 3:** Percentage of a structure on the reCTs that falls within the volume of the structure on the pCT, and within the isotropically expanded structure, for 90% of the patients. In bold and italic marked the expansion for which the percentage is above 95%, and in the most right column the corresponding expansion in mm.

Structure	Isotropic margin [mm]						Expansion needed for 90 % of patients to cover 95 % [mm]
	*0*	*1*	*2*	*3*	*4*	*5*	
Cornea	44.0	73.5	85.5	92.4	***97.3***	99.9	4
Lacrimal gland	61.7	89.2	94.3	***98.1***	99.1	99.9	3
Lens	25.7	46.1	65.6	85.1	***97.3***	100	4
Macula	1.4	21.6	50.0	83.8	***100***	100	4
Oculus	90.0	***98.4***	100	100	100	100	1
Optic nerve	63.0	81.3	90.0	***96.8***	99.7	100	3
Optic nerve cranial	69.0	92.4	***98.5***	100	100	100	2
Optic nerve orbital	54.5	76.4	86.9	***96.1***	99.7	100	3
Optic nerve orbital distal	68.1	90.3	***99.3***	100	100	100	2
Optic nerve orbital proximal	31.7	57.7	78.2	92.5	***97.5***	99.7	4
Retina	57.7	81.8	89.9	***95.8***	99.5	99.9	3

### 3.2 Eye open or closed

Eyelid status open was seen for 5/20 (25 %) pCT-scans and 40/100 (40 %) reCT-scans. For 13 patients at least one CT-scan with eyes open was found, for 7 of these patients at least 4 out of 6 CT-scans were with eyes open. Three patients had their eyes open during all CT-scans, seven patients closed their eyes during all CT-scans.

**Fig. 2:**
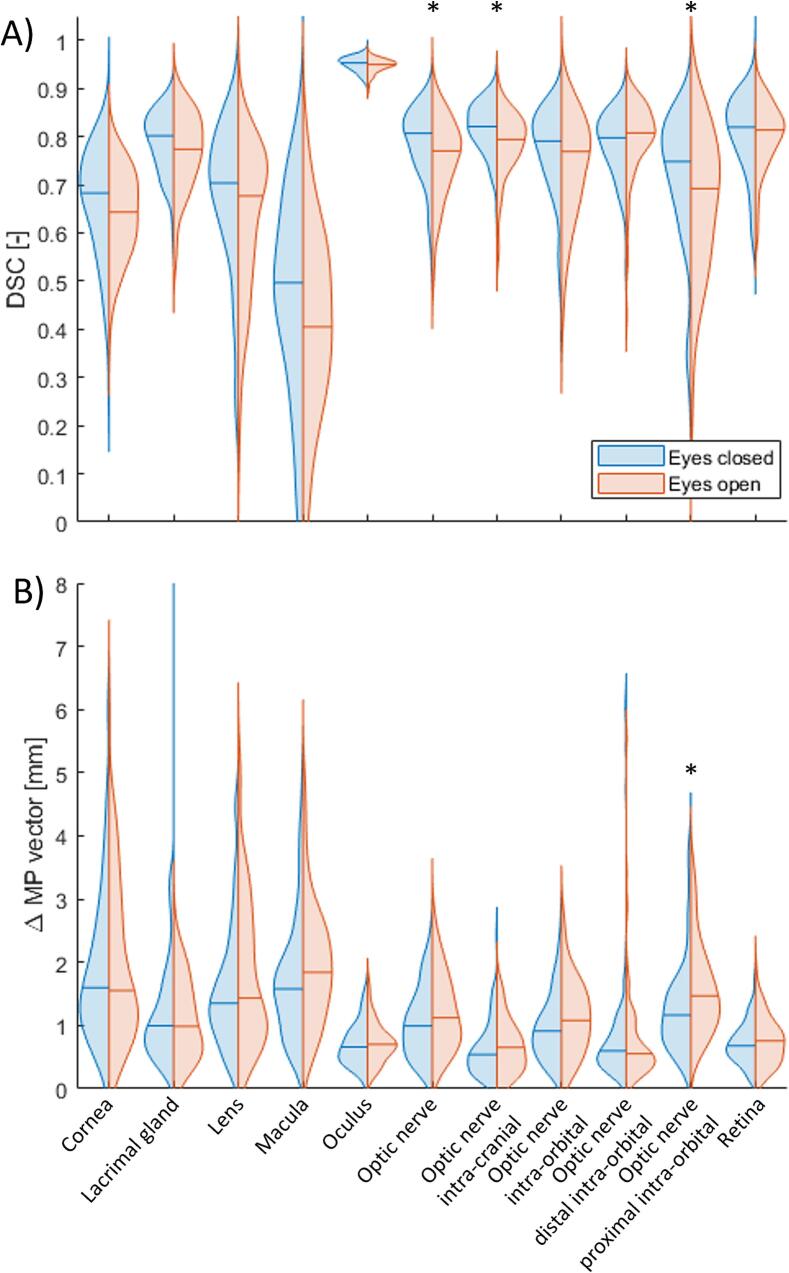
A) DSC and B) ΔMP vector results, comparing eyes closed vs. eyes open. * = statistically significant difference eyes closed vs. eyes open (Mann-Whitney *U* test, p < 0.0045). Horizontal lines represent the median values. DSC = Dice Similarity Coefficient, MP = midpoint.

Statistically significant higher DSC values were found for reCTs with eyes closed vs. open for the ON, intra-cranial ON, and proximal intra-orbital ON, see Supplementary Material Table S4. For the ΔMP vector, a statistically significant higher value for eyes open vs. closed was found for the proximal intra-orbital ON. Fig. 2 shows DSC and ΔMP vector results comparing the reCTs with eyes closed vs. open. For the AD per direction, a statistically significant difference eyes closed vs. open was found for some structures, see Supplementary Material Table S5. The AD in the separate directions was higher for the subgroup reCTs with eyes open. However, these differences were only submillimeter.

### 3.3 Dose-volume analysis

**Fig. 3:**
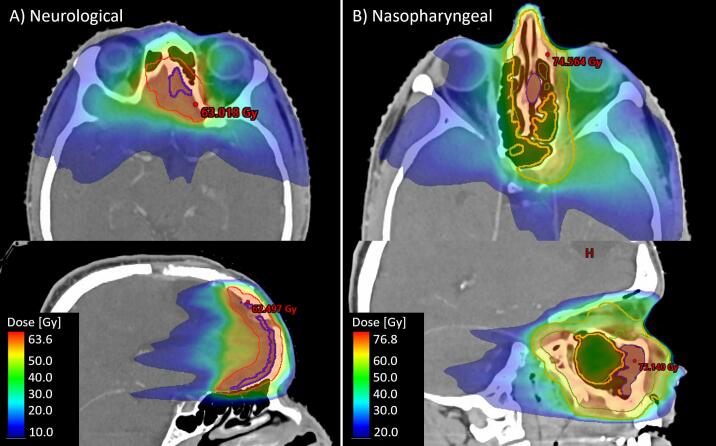
A) Dose distribution in the transversal and sagittal plan of the neurological treatment plan, and B) of the nasopharyngeal treatment plan.

Dose distributions of the neurological and nasopharyngeal plan were shown in Fig. 3. When comparing D_mean_ on the pCT to D_mean_ on any of the reCTs, highest absolute dose difference in the neurological plan was found for the left lens (pCT: 8.8 Gy, reCT: 19.1 Gy). Highest percentual difference was found for the right cornea (pCT: 2.2 Gy, reCT: 5.1 Gy, +132 %). In the nasopharyngeal plan, highest absolute and percentual dose difference in D_mean_ was found for the right lens (pCT: 26.6 Gy, reCT: 29.7 Gy, +12 %).

In the neurological plan, the highest increasing absolute dose difference in D_0.03cm3_ was found for the left lens (pCT: 13.2 Gy, reCT: 22.9 Gy) Highest percentual difference was found for the right cornea (pCT: 5.2 Gy, reCT: 13.9 Gy, +167 %). In the nasopharyngeal plan, highest absolute and percentual dose difference in D_0.03cm3_ was found for the left lacrimal gland (pCT: 32.3 Gy, reCT: 39.2 Gy, +21 %).

In general, dose-volume differences were higher for the neurological plan compared to the nasopharyngeal plan (see Supplementary Material Table S6).

## 4 Discussion

In this study, the movement of optical structures was quantified over the course of RT, by determining the position of these optical structures on CT-scans at different moments during the course of treatment. It was shown that all optical structures can move to a certain extent when in a natural position during CT-scanning, towards a maximum of a couple of millimeters. This indicates that natural movement, as will occur without gazing instructions, should be investigated in more detail and potentially considered in treatment planning.

The cornea showed the largest movement in ΔMP, especially in Y-direction. For the ON subregions, highest median ΔMP vector was found for the proximal intra-orbital ON (1.3 mm). For the AD, the ON presented with the highest median AD in the negative Z-direction (3.0 mm). Overall for the intra-orbital subregions of the ON a larger AD was found compared with the intra-cranial subregion. This intra-cranial ON is enclosed by the cranial orbital channel, thus allowing less movement. Considering the ΔMP of the oculi as control, a 0.7 mm vector could be subtracted from all other structures to eliminate some inaccuracies. AD greater than 2 mm was observed in 84 % of reCTs for the lacrimal gland, which shows that structures other than the ON and cornea also exhibit relevant movement and should be considered. The direction of motion of the lacrimal gland was studied and found to be mainly in the anterior-posterior (Z) plane, which corresponds to the results found here [10].

A recent study by Hoeben et al. evaluated the movement of the lenses, orbital optic nerves, and optic discs during craniospinal irradiation, to determine its maximum position laterally and vertically from original neutral gaze position [11]. They found greatest lateral and vertical movement being 6.7 and 9.0 mm for optic discs. This investigation was recreated with a focus on the anterior section of the optic nerve; a maximum lateral and vertical movement of 6.6 and 7.0 mm was found from center position [12]. The results of these studies prove that there is significant movement of optical structures possible from an assumed neutral gaze position. However, their focus on establishing the largest possible movement fails to encapsulate what happens naturally during RT and whether such movement is clinically relevant. Geneser et al. reported an average movement of the left and right lens of respectively 1.6 and 1.7 mm, and a maximum movement of 3.6 and 4.7 mm, determined on daily CBCT-imaging without gazing instructions [8]. In our study we have shown that this movement also occurred during CT-imaging, and that optical structures other than the lens and optic nerves exhibit similar movements. If such movement is observed during CT-scanning and RT, it strongly suggests that relying on the initial position for treatment planning does not account for the potential movement during treatment delivery. To include this movement in treatment planning, a hypothetical PRV-like expansion was determined needed to cover 95 % of the volume of the optical structures on the reCTs. An expansion of 4 mm was needed for the cornea, lens, macula, and proximal intra-orbital ON. Only CT-recorded positions were used in the current analysis, which potentially introduced a bias towards looking in the forward direction. During treatment on a C-arm linac, gantry rotations could negatively influence eye movements, resulting in more extreme positions than were registered using CT-scans. However, this study presents a good approximation of the natural positions during treatment, better than other studies where maximum gazing positions were analyzed. Gazing instructions could be beneficial in decreasing this movement and minimizing these extreme positions. This should be validated using daily CBCT-imaging analyses.

The eyelid status being open or closed did have an influence on the movement of certain structures. For the vector ΔMP, a statistically significant difference eyes closed vs. open was found for the macula, intra-orbital ON, and proximal intra-orbital ON, with a higher ΔMP vector for the reCTs with eyes open, indicating more movement. Thus, movement of especially the macula and the ON will be influenced by open eyelid status. For the AD, the (proximal) intra-orbital ON showed a statistically significant difference in AD between eyelid status for several directions, but these were only submillimeter. Movement of especially the intra-orbital region of the ON, specifically the proximal subregion, is potentially most sensitive to open eyelid status. Gazing instructions might decrease the movement of optical structures or could potentially even decrease the dose to certain optical structures when instructions would point the gaze in opposite direction. On the other hand, gazing instructions might be difficult during CT-scanning or during treatment on a C-arm linac due to movement of the equipment. However, there are some eye fixation aids that could be helpful, e.g. a small fixation light attached to a mask system or a marker placed on a plexiglass fixture [13,14]. Another option would be to ask the patient to close their eyes during the imaging and treatment.

The dose-volume analysis showed that dose differences due to optical movement might be higher for neurological compared to nasopharyngeal treatment plans. This depends strongly on the location of the tumor volume and prescribed dose. In our proof-of concept we observed a considerable influence on dose received by the optical structures because of inter-fraction movement. However, during treatment this influence could eventually even out as a result of hyperfractionation and thus different optical positions for each fraction. More research and expanding this analysis would be recommended to validate these findings and to be able to conclude about clinical relevance. Gazing instructions might even become imperative when an optical structure is located close to the target. Azemar et al. studied the influence of gaze direction during head-and-neck RT of paraoptic tumors and they found a dose difference of ≥5 Gy for the retina and macula for at least one CT-scan, which could be related to a systematic gaze angle deviation [15]. This shows that there indeed can be dosimetric implications of different eye positions.

This study had some limitations. The accuracy and consistency of RT planning are influenced by e.g. inter-observer variability in contouring, registration uncertainty, and set-up variation within masks. Contouring variability remains a significant challenge in radiotherapy, which can lead to differences in delineation of target volumes and OARs, potentially affecting treatment outcomes. To mitigate this, standardized contouring protocols and training are essential to enhance consistency [9,[16], [17], [18], [19]]. In this study, there was no weekly MRI available, limiting the accuracy of delineation for e.g. the optic nerves as specified in the EPTN contouring atlas [9]. We calculated isotropic expansions for all optical structures based on 95 % volume coverage over all patients. However, for some structures anisotropic expansions or a margin calculation using the van Herk formula might be a better approach [20]. The dose-volume influence of this additional margin was considered outside the scope of this study. We did not perform an inter- or intra-patient eye movement analysis due to the limited amount of data. Additionally, the results could be different for patients with a tumor related to the optical structures, e.g. an optic glioma or meningioma.

The observed movement of optical structures during reCTs in most patients underscores the necessity of incorporating a PRV-margin in clinical practice to accommodate this variability, especially in cases where a high dose to this structure is expected. To fully understand the dose-volume implications of these movements, further analysis is essential. Additionally, future research should explore the potential of implementing gaze instructions to determine if this can reduce positional discrepancies observed. Closing the eyes during imaging and treatment sessions will already decrease some movement.

## CRediT authorship contribution statement

**Femke Vaassen:** Conceptualization, Data curation, Methodology, Investigation, Visualization, Writing – original draft. **David Hofstede:** Data curation, Methodology, Visualization, Writing – review & editing. **Nikolina Birimac:** Data curation. **Inge Compter:** Writing – review & editing. **Marlies Granzier:** Formal analysis, Writing – review & editing. **Wouter van Elmpt:** Supervision, Writing – review & editing. **Catharina M.L. Zegers:** Conceptualization, Supervision, Visualization, Writing – review & editing. **Daniëlle B.P. Eekers:** Conceptualization, Supervision, Visualization, Writing – review & editing.

## Declaration of competing interest

The authors declare that they have no known competing financial interests or personal relationships that could have appeared to influence the work reported in this paper.
