# Supplementary material for: Quantifying movement of optical structures during radiotherapy treatment for tumors near the orbita
Source: Phys Imaging Radiat Oncol. 2025 Aug 30;35:100830. doi: 10.1016/j.phro.2025.100830 (PMC12446562; doi:10.1016/j.phro.2025.100830)
Supplement: Supplementary Data 1 [file mmc1.pdf]

SUPPLEMENTARY MATERIAL

Quantifying movement of optical structures during radiotherapy treatment for tumors near the orbita

Table S1 – Overview of patient characteristics (n = 20, number of scans = 120).

Characteristic	Value
Gender	
Male	8 (40%)
Female	12 (60%)
Age at start RT (in years)	
Mean	46
Median	48
Range	19-66
Tumor type	
Oligodendroglioma	12 (60%)
Astrocytoma	5 (25%)
Meningioma	1 (5%)
Mesenchymal chondrosarcoma	1 (5%)
Hemangiopericytoma	1 (5%)
Eyelid status on CT scan	
Open	45 (37.5%)
Closed	75 (62.5%)

Table S2 – Overview of Dice Similarity Coefficient (DSC) results, reported as median (range) after first averaging over the five reCTs per patient.

Structure	DSC [-]
Cornea	0.65 (0.54-0.78)
Lacrimal gland	0.78 (0.61-0.87)
Lens	0.66 (0.44-0.82)
Macula	0.46 (0.16-0.71)
Oculus	0.95 (0.91-0.97)
Optic nerve	0.78 (0.65-0.86)
Optic nerve intra-cranial	0.80 (0.71-0.88)
Optic nerve intra-orbital	0.77 (0.59-0.87)
Optic nerve distal intra-orbital	0.80 (0.64-0.87)
Optic nerve proximal intra-orbital	0.70 (0.46-0.85)
Retina	0.80 (0.70-0.90)

Table S3 – Percentage of reCTs with absolute difference (AD) greater than 2 mm, per structure.

Structure	% reCTs
Cornea	79
Lacrimal gland	84
Lens	54
Macula	52
Oculus	57
Optic nerve	73

Optic nerve intra-cranial	19
Optic nerve intra-orbital	77
Optic nerve distal intra-orbital	45
Optic nerve proximal intra-orbital	65
Retina	52

Table S4 – Overview of statistical tests comparing results for reCTs where patients has their eyes closed vs. open. Results are shown for DSC and the ΔMP vector. Values are presented as median (range) over all reCTs, and p-values were determined using a Mann-Whitney U-test. Bold values represent a p-value < 0.0045.

**Structure**	**DSC [-]**			**ΔMP [mm]**		
	Eyes closed	*Eyes open*	*p-value*	*Eyes closed*	*Eyes open*	*p-value*
Cornea	0.68 (0.28-0.88)	0.64 (0.4-0.78)	0.023	1.6 (0.2-6.1)	1.9 (0.4-5.9)	0.749
Lacrimal gland	0.8 (0.57-0.88)	0.77 (0.55-0.88)	0.140	1.0 (0.2-9.1)	1.0 (0.1-2.7)	0.621
Lens	0.7 (0.22-0.91)	0.68 (0.15-0.88)	0.020	1.4 (0.2-4.6)	1.4 (0.3-5.1)	0.320
Macula	0.5 (0-0.83)	0.41 (0-0.77)	0.026	1.6 (0.4-5)	1.8 (0.5-5.0)	0.027
Oculus	0.95 (0.91-0.98)	0.95 (0.89-0.98)	0.077	0.7 (0.1-1.6)	0.7 (0.2-1.7)	0.377
Optic nerve	0.81 (0.53-0.89)	0.77 (0.5-0.91)	**0.003**	1.0 (0.2-2.5)	1.1 (0.3-2.8)	0.050
Optic nerve intra-cranial	0.82 (0.65-0.91)	0.79 (0.56-0.9)	**0.000**	0.5 (0.1-2.4)	0.7 (0.1-1.7)	0.193
Optic nerve intra-orbital	0.79 (0.38-0.89)	0.77 (0.41-0.91)	0.024	0.9 (0.1-2.8)	1.1 (0.2-2.7)	0.041
Optic nerve distal intra-orbital	0.8 (0.53-0.9)	0.81 (0.43-0.91)	0.127	0.6 (0.1-6.1)	0.6 (0.1-5.5)	0.615
Optic nerve proximal intra-orbital	0.75 (0.24-0.89)	0.69 (0.19-0.89)	**0.003**	1.2 (0.2-4.0)	1.5 (0.5-3.6)	**0.001**
Retina	0.82 (0.57-0.97)	0.81 (0.59-0.92)	0.498	0.7 (0.1-1.8)	0.8 (0.1-1.9)	0.498

Table S5 – Overview of statistical tests comparing results for reCTs where patients has their eyes closed vs. open for the AD in the six different directions. Values are presented as median (range) over all reCTs, and p-values were determined using a Mann-Whitney U-test. Bold values represent a p-value < 0.0045.

**Structure**	**X- [mm]**			**X+ [mm]**		
	Eyes closed	*Eyes open*	*p-value*	*Eyes closed*	*Eyes open*	*p-value*
Cornea	0.2 (-0.4-0.6)	0.2 (-0.2-0.5)	**0.003**	0.2 (-0.3-0.9)	0.2 (-0.3-0.8)	0.049
Lacrimal gland	0.1 (0.0-0.4)	0.1 (0.0-0.4)	0.834	0.1 (0.0-8.6)	0.1 (0.0-0.4)	0.480
Lens	0.1 (-0.4-0.4)	0.1 (-0.2-0.4)	0.830	0.1 (-0.2-0.6)	0.1 (-0.2-0.5)	0.645
Macula	0.1 (-0.2-0.4)	0.1 (-0.2-0.3)	0.682	0.0 (-0.4-0.3)	0.1 (-0.2-0.3)	0.130
Oculus	0.1 (-0.1-0.4)	0.2 (0.0-0.5)	0.020	0.1 (-0.1-0.5)	0.1 (-0.1-0.6)	0.784
Optic nerve	0.1 (-0.1-1.5)	0.1 (0.0-2.2)	0.414	0.1 (-0.3-1.8)	0.2 (-0.1-1.9)	0.018
Optic nerve intra-cranial	0.0 (-0.1-0.5)	0.0 (-0.2-0.4)	0.816	0.1 (-0.1-0.4)	0.0 (-0.2-0.5)	0.229
Optic nerve intra-orbital	0.1 (-0.1-1.0)	0.1 (-0.1-0.9)	0.293	0.1 (-0.3-1.0)	0.1 (-0.2-0.9)	0.035
Optic nerve distal intra-orbital	0.0 (-0.2-0.3)	0.0 (-0.1-0.4)	0.106	0.0 (-0.1-0.6)	0.0 (-0.1-0.6)	0.968
Optic nerve proximal intra-orbital	0.1 (-0.1-0.8)	0.1 (-0.1-0.6)	0.437	0.1 (-0.3-0.3)	0.1 (-0.2-0.7)	0.016
Retina	0.1 (-0.1-0.4)	0.2 (0.0-0.5)	0.010	0.1 (-0.1-0.5)	0.1 (-0.1-0.6)	0.459
**Structure**	**Y- [mm]**			**Y+ [mm]**		
	Eyes closed	*Eyes open*	*p-value*	*Eyes closed*	*Eyes open*	*p-value*
Cornea	0.0 (-0.6-0.3)	0.1 (-0.2-0.4)	**0.000**	-0.1 (-0.6-0.5)	-0.1 (-0.7-0.3)	0.090

Lacrimal gland	0.0 (-0.8-0.4)	0.0 (-0.4-0.3)	0.007	0.0 (-0.3-0.2)	0.0 (-0.2-0.3)	0.839
Lens	0.0 (-0.4-0.4)	0.1 (-0.2-0.5)	0.005	0.0 (-0.4-0.5)	-0.1 (-0.5-0.2)	**0.000**
Macula	-0.1 (-0.3-0.4)	-0.1 (-0.5-0.3)	0.157	0.0 (-0.5-0.2)	0.0 (-0.4-0.4)	0.827
Oculus	0.0 (-0.2-0.2)	0.0 (-0.1-0.2)	**0.001**	0.0 (-0.2-0.2)	0.0 (-0.2-0.1)	0.027
Optic nerve	0.0 (-0.3-0.3)	0.0 (-0.3-0.1)	0.151	0.0 (-0.3-0.2)	0.1 (-0.2-0.5)	**0.002**
Optic nerve intra-cranial	0.0 (-0.1-0.1)	0.0 (-0.1-0.1)	0.025	0.0 (-0.1-0.1)	0.0 (-0.1-0.1)	0.026
Optic nerve intra-orbital	0.0 (-0.3-0.3)	0.0 (-0.3-0.1)	0.150	0.0 (-0.4-0.2)	0.0 (-0.2-0.5)	0.085
Optic nerve distal intra-orbital	0.0 (-0.3-0.1)	0.0 (-0.2-0.1)	0.403	0.0 (-0.2-0.1)	0.0 (-0.1-0.2)	0.021
Optic nerve proxi-mal intra-orbital	0.0 (-0.8-0.4)	-0.1 (-0.5-0.1)	0.016	0.0 (-0.5-0.5)	0.0 (-0.3-0.5)	0.085
Retina	0.0 (-0.2-0.2)	0.0 (-0.2-0.2)	0.029	0.0 (-0.2-0.2)	0.0 (-0.2-0.1)	**0.002**
**Structure**	**Z- [mm]**			**Z+ [mm]**		
	Eyes closed	*Eyes open*	*p-value*	*Eyes closed*	*Eyes open*	*p-value*
Cornea	0.1 (-0.1-0.6)	0.1 (0.0-0.5)	0.266	0.1 (-0.1-0.3)	0.1 (0.0-0.4)	**0.000**
Lacrimal gland	0.2 (0.0-1.0)	0.2 (-0.1-0.9)	0.173	0.2 (-0.1-1.0)	0.2 (0.0-0.6)	0.112
Lens	0.1 (-0.1-0.3)	0.1 (-0.1-0.3)	0.658	0.0 (-0.1-0.1)	0.1 (-0.1-0.2)	0.008
Macula	0.0 (-0.1-0.3)	0.0 (-0.1-0.2)	0.845	0.0 (-0.2-0.3)	0.1 (-0.1-0.3)	0.027
Oculus	0.2 (0.0-0.5)	0.1 (0.0-0.5)	0.618	0.1 (0.0-0.6)	0.2 (0.0-0.4)	**0.000**
Optic nerve	0.2 (-0.1-3.2)	0.3 (0.0-2.4)	0.129	0.1 (-0.1-2.7)	0.2 (-0.1-3.1)	**0.003**
Optic nerve intra-cranial	0.1 (-0.1-0.8)	0.1 (-0.2-0.4)	0.901	0.1 (-0.1-0.8)	0.1 (-0.1-0.5)	0.983
Optic nerve intra-orbital	0.1 (-0.1-2.0)	0.3 (-0.1-1.9)	0.009	0.1 (-0.1-2.4)	0.2 (-0.1-2.1)	0.021
Optic nerve distal intra-orbital	0.1 (-0.1-1.1)	0.1 (-0.1-0.9)	0.297	0.1 (-0.1-1.2)	0.1 (-0.3-0.7)	0.250
Optic nerve proximal intra-orbital	0.1 (-0.2-1.2)	0.1 (-0.1-1.5)	0.059	0.1 (-0.1-1.3)	0.1 (-0.1-1.2)	**0.001**
Retina	0.1 (0.0-0.5)	0.1 (0.0-0.5)	0.671	0.1 (-0.1-0.6)	0.2 (0.0-0.4)	0.006

Table S6 – Dose-volume results for both the neurological and the nasopharyngeal plan. Results are reported for the mean dose (D_{mean}) and the dose to 0.03 cm^3 ($D_{0.03cm3}$) of the volume, as the dose on the pCT and between brackets the range of doses on the reCTs. For the macula, $D_{0.03cm3}$ was not reported because the volume of the structure was below 0.03 cm^3 for most CT scans.

**Structure**	**Neurological**		**Nasopharyngeal**	
	D_{mean} *[Gy]*	$D_{0.03cm3}$ *[Gy]*	D_{mean} *[Gy]*	$D_{0.03cm3}$ *[Gy]*
Cornea left	7.4 (7.6-16.0)	19.0 (17.7-27.9)	32.2 (32.0-33.8)	36.6 (35.7-37.6)
Cornea right	2.2 (2.3-5.1)	5.2 (5.0-13.9)	29.6 (29.4-31.0)	33.9 (33.8-35.8)
Lacrimal gland left	13.6 (12.6-15.0)	24.1 (18.7-24.8)	28.2 (27.3-29.5)	32.3 (30.1-39.2)
Lacrimal gland right	5.1 (5.3-7.4)	15.6 (16.9-23.2)	18.7 (18.8-19.8)	22.6 (23.0-26.3)
Lens left	8.8 (8.6-19.1)	13.2 (12.8-22.9)	35.6 (35.0-35.8)	36.3 (35.7-36.6)
Lens right	2.1 (2.1-4.3)	2.4 (2.4-5.9)	26.6 (27.3-29.7)	27.9 (28.9-31.0)
Macula left	15.3 (7.8-15.7)	-	46.4 (44.4-49.3)	-
Macula right	5.5 (2.5-6.1)	-	26.1 (23.9-26.5)	-
Oculus left	16.2 (16.2-17.3)	45.6 (44.8-48.0)	38.5 (38.3-38.7)	52.3 (51.5-52.5)
Oculus right	5.7 (5.8-6.4)	31.3 (30.2-34.3)	27.5 (27.6-28.0)	38.9 (39.3-40.4)
Optic nerve left	20.0 (12.0-23.0)	28.6 (19.2-28.4)	54.3 (53.5-54.9)	56.3 (54.5-56.2)
Optic nerve intra-cranial left	23.2 (22.6-23.0)	26.0 (24.7-26.5)	54.5 (54.7-54.9)	56.0 (55.9-56.1)
Optic nerve intra-orbital left	19.1 (12.0-19.7)	27.7 (19.2-27.9)	54.3 (53.5-54.4)	55.8 (54.5-55.7)
Optic nerve distal intra-orbital left	19.2 (17.3-19.6)	27.7 (22.2-26.9)	54.3 (54.0-54.3)	55.5 (54.5-55.3)
Optic nerve proximal intra-orbital left	18.9 (12.0-19.7)	23.9 (19.2-25.3)	54.3 (53.5-54.4)	55.3 (55.2-55.3)
Optic nerve right	16.5 (7.7-18.1)	28.8 (14.3-28.3)	33.8 (31.6-35.6)	37.8 (35.0-37.6)

Optic nerve intra-cranial right	17.3 (16.9-17.4)	19.4 (18.5-19.2)	33.5 (33.6-33.8)	35.1 (35.0-35.3)
**Optic nerve intra-orbital right**	16.4 (7.7-18.1)	28.8 (14.3-28.3)	33.9 (31.6-35.6)	37.8 (35.3-37.6)
**Optic nerve distal intra-orbital right**	18.4 (17.6-18.1)	28.4 (25.1-25.9)	35.6 (35.4-35.6)	37.8 (37.1-37.3)
**Optic nerve proximal intra-orbital right**	14.7 (7.7-14.5)	24.4 (14.3-24.0)	32.5 (31.6-33.4)	36.0 (35.3-36.0)
**Retina left**	17.6 (17.4-17.9)	45.6 (45.0-48.2)	40.4 (40.0-40.5)	52.3 (51.4-52.4)
**Retina right**	7.9 (7.8-8.5)	31.3 (29.9-33.9)	28.2 (28.0-28.6)	38.9 (39.2-40.4)
